# Identifying tripartite relationship among cortical thickness, neuroticism, and mood and anxiety disorders

**DOI:** 10.1038/s41598-024-59108-1

**Published:** 2024-04-11

**Authors:** Renata Rozovsky, Michele Bertocci, Satish Iyengar, Richelle S. Stiffler, Genna Bebko, Alexander S. Skeba, Tyler Brady, Haris Aslam, Mary L. Phillips

**Affiliations:** 1 Department of Psychiatry, University of Pittsburgh School of Medicine, University of Pittsburgh, 302 Loeffler Building, 121 Meyran Ave., Pittsburgh, PA USA; 2 Department of Statistics, University of Pittsburgh, Pittsburgh, PA USA

**Keywords:** Neuroticism, Anxiety, Depression, Mania, Hypomania, Cortical thickness, Mediation, Psychology, Prefrontal cortex, Biomarkers

## Abstract

The number of young adults seeking help for emotional distress, subsyndromal-syndromal mood/anxiety symptoms, including those associated with neuroticism, is rising and can be an early manifestation of mood/anxiety disorders. Identification of gray matter (GM) thickness alterations and their relationship with neuroticism and mood/anxiety symptoms can aid in earlier diagnosis and prevention of risk for future mood and anxiety disorders. In a transdiagnostic sample of young adults (n = 252;177 females; age 21.7 ± 2), Hypothesis (H) 1*:*regularized regression followed by multiple regression examined relationships among GM cortical thickness and clinician-rated depression, anxiety, and mania/hypomania; H2:the neuroticism factor and its subfactors as measured by NEO Personality Inventory (NEO-PI-R) were tested as mediators. Analyses revealed positive relationships between left parsopercularis thickness and depression (B = 4.87, *p* = 0.002), anxiety (B = 4.68, *p* = 0.002), mania/hypomania (B = 6.08, *p* ≤ 0.001); negative relationships between left inferior temporal gyrus (ITG) thickness and depression (B = − 5.64, *p* ≤ 0.001), anxiety (B = − 6.77, *p* ≤ 0.001), mania/hypomania (B = − 6.47, *p* ≤ 0.001); and positive relationships between left isthmus cingulate thickness (B = 2.84, *p* = 0.011), and anxiety. NEO anger/hostility mediated the relationship between left ITG thickness and mania/hypomania; NEO vulnerability mediated the relationship between left ITG thickness and depression. Examining the interrelationships among cortical thickness, neuroticism and mood and anxiety symptoms enriches the potential for identifying markers conferring risk for mood and anxiety disorders and can provide targets for personalized intervention strategies for these disorders.

## Introduction

The number of young adults seeking help from mental health professionals for emotional distress^1,2^, including a range of subsyndromal to syndromal mood (depression, mania/hypomania) and anxiety symptoms, functional disabilities, and behavioral problems, is rising dramatically. Identifying objective neural markers of risk for the range of subsyndromal to syndromal mood and anxiety symptoms will not only aid earlier identification of risk for developing mood and anxiety disorders, but will also provide valuable insights into underlying neural mechanisms and provide targets to guide future interventions. It is well established that the personality trait neuroticism wields substantial public health implications^3,4^ and is closely related to mood and anxiety disorders^5–17^. One way forward to identifying objective neural markers of risk for mood and anxiety disorders is thus to identify tripartite relationships among neural markers relevant to mood and anxiety disorders, including those supporting emotional perception and regulation, and: neuroticism and subsyndromal-syndromal mood and anxiety symptom severity.

Gray matter (GM) measures show good test–retest reliability^18–21^, are critical structural underpinnings of neural activity, and, as such, are promising neural markers of neuroticism and predictors of future mood and anxiety disorders, as measured by subsyndromal-syndromal mood and anxiety symptom severity. Previous studies in adults have provided mixed results, however, with numerous studies demonstrating widespread decreases in cortical thicknesses, particularly in the prefrontal, cingulate, temporal, and parietal cortices in individuals with^22–39^ and at risk of^40–44^ mood and anxiety disorders, while some studies indicate cortical thickening, related to these disorders^28,34,45–48^. The mixed nature of these findings suggests that the relationship between cortical thickness and mood and anxiety might be driven by other factors such as personality traits, specifically by neuroticism, but these relationships remain to be examined.

While an expanding body of research revealed associations between neuroticism and GM volumes, predominantly in the prefrontal and cingulate cortices^49–54^, some studies have examined relationships between neuroticism and cortical thickness in key emotion regulation GM regions in adult populations. In healthy individuals, neuroticism has been positively associated with cortical thickness in left frontal and parietal cortices^55–57^, with both increased and decreased thickness reported in right frontal and middle temporal cortex, and the left pars opercularis^52,56–59^. Together, these findings suggest that cortical thickness abnormalities are associated with higher levels of neuroticism, but a mechanistic understanding of the relationships between GM thickness, neuroticism, and clinical symptoms is needed. This is especially important during young adulthood —a critical period for the onset of mental health issues and sensitive to interventions that can take advantage of the plasticity of the brain, given the continued neurodevelopment during this period^60–63^.

Studies of neuroticism have also shown relationships with mood and anxiety disorders^5–17^, indeed, the neuroticism domain of the Revised NEO Personality Inventory (NEO PI-R)^64^ encompasses subfactors such as “anxiety” and “depression”. These subfactors however, differ from the metrics provided by symptom level measures such as the Hamilton Anxiety Rating Scale (HAMA)^65^ and the Hamilton Rating Scale for Depression (HRSD)^66^ suggesting that they assess inherently different constructs. HAMA and HRSD evaluate the respondent’s state over the seven days preceding the clinical interview, focusing on both the physical and cognitive aspects of anxiety and depression respectively. In contrast, the NEO PI-R measures trait emotional experiences over the lifespan. Given these fundamental differences in measurement and the close relationship of neuroticism to mood and anxiety psychopathology, a careful exploration of the relationships among neuroticism, its respective subfactors, and the clinician-administered Hamilton Depression, Anxiety, and Mania scales is a logical step in understanding these tripartite relationships.

Studies using mediation analyses^67,68^ to examine the nature of tripartite relationships among GM, personality traits and behavior reported that neuroticism partially mediated the positive relationship between GM volume in the left dorsolateral prefrontal cortex, an emotional regulation region, and loneliness^69^; and neuroticism mediated the positive relationship between cortical thickness of the superior frontal cortex, an emotion processing region, and aggressive behavior^70^. No study to our knowledge, however, examined tripartite relationships among cortical thickness, neuroticism and subsyndromal-syndromal mood and anxiety symptom severity. While cross-sectional designs cannot infer causality, the temporal relationship among GM development, neuroticism, and clinical symptoms might suggest directionality in the relationships among these factors. Specifically, the human brain reaches 90% of its full size by five years of age, and GM cortical thickness shows both blooming and pruning during childhood and adolescence, with stable measures reached in adulthood^71^. Neuroticism also develops in childhood^72–74^ and becomes more stable across adulthood^75–80^, while mood and anxiety disorders most commonly develop in young adulthood^63,81,82^. It is thus possible that an individual’s early life trajectory, in conjunction with specific brain characteristics, may lead to the development of neuroticism, which in turn plays an important role in the emergence of mood and anxiety symptoms. While the directionality of relationships among GM, personality trait, and mood and anxiety symptoms remains to be clarified, the reported relationships between GM and the relatively more temporally stable neuroticism trait, and between GM and relatively more rapidly changing mood and anxiety symptoms, motivated our choice of neuroticism and its subfactors as mediators of GM-mood and anxiety symptom relationships.

Our study adopted a transdiagnostic approach, recruiting young adults who exhibited a spectrum of subsyndromal to syndromal mood and anxiety symptoms to identify the relationships among GM cortical thickness, neuroticism, and mood and anxiety symptom severity. This approach sought to transcend conventional diagnostic boundaries, offering profound insights into these relationships ^83–85^ and aimed to fill an important mechanistic gap in the literature concerning these relationships. Our hypotheses, grounded in existing literature generally showing reduced cortical thickness decrease in mood and greater cortical thickness in anxiety disorders were:

1. Reduced cortical thickness within prefrontal and temporal regions would be associated with greater severity of mood symptoms, specifically depression and mania/hypomania, whereas greater cortical thickness would be associated with greater severity of anxiety symptoms.
2. In the tripartite relationship, neuroticism and its specific subfactors, namely anxiety, anger/hostility, depression, self-consciousness, impulsiveness, and vulnerability would mediate the relationship between cortical thickness in specified regions and symptom severity.

## Methods

### Participants

Neuroimaging and clinical assessment data were employed from an ongoing study of young adults (18–25-year-olds) who were recruited across a range of mood and anxiety symptom severity (R37MH100041). 269 young adults 18–25 years were recruited from the general population via advertisement: 136 were seeking treatment for psychological distress and 133 were healthy. This approach ensured that a range of subsyndromal-syndromal mood and anxiety symptom severity was represented in recruited participants. From an initial sample, 17 participants were excluded due to: missing clinical data (n = 1), errors in segmentation/parcellation processing (n = 5), taking psychotropic (antidepressants) medications (n = 11), due to their possible influence on GM measures. The final sample comprised 252 individuals (123 seeking help for emotional distress, 129 non-distressed, healthy controls, mean age 21.7 ± 2 years, 177 female; Table 1). In the distressed group, 39 participants were currently diagnosed with depressive and 75 with anxiety disorders (including 26 participants who had both diagnoses). All participants were right-handed and English-speaking. The study protocol was approved by the University of Pittsburgh Institutional Review Board, all research was performed in accordance with relevant guidelines/regulations^86^, and all participants provided informed consent.

**Table 1:** Demographic and clinical characteristics of final sample (n = 252, 169 distressed, 129 healthy).

	Distressed	Healthy
Age	21.6 ± 2.1	21.7 ± 1.9
Sex f/m	87/36	90/39
IQ mean ± SD	108.2 ± 8.0	108.3 ± 6.5
Education mean ± SD	5.3* ± 1.1	5.5* ± 1.1
Ethnicity Hispanic or Latino	4	3
Race AA/white/Asian/MTOR	19/78/20/6	10/87/28/4
Self-reported smoking	13	8
HAMA mean score ± SD, min/max	12.23 ± 6.0, 0/27	0.84 ± 1.3, 0/7
HRSD mean score ± SD, min/max	14.99 ± 6.2, 1/30	1.18 ± 2.0, 0/13
YMRS mean score ± SD, min/max	2.65 ± 1.8, 0/8	0.28 ± 0.9, 0/8
NEO Neuroticism mean score ± SD, min/max	116.24 ± 20.7, 59/170	74.61 ± 17.2, 33/108

3 participants had a HAMA score  > 25 (moderate to severe), 23 participants had scores 18–24 (mild to moderate), and 226 had scores  < 17 (none or mild severity). For HRSD 17 participants had scores  > 23 (severe to very severe), 19 participants had scores 19–22 (moderate to severe), 36 participants had scores 14–18 (mild to moderate), 40 participants had scores 8–13 (subthreshold to mild), and 140 participants had scores 0–7 (not depressed). For YMRS the scores were  < 9 for all participants.

MTOR, more than one race; HAMA, Hamilton Anxiety Rating Scale; HRSD, Hamilton Rating Scale for Depression; YMRS, Young Mania Rating Scale; NEO, NEO PI-R Five Factor Inventory.

*at least one year of education post high school.

### Assessment of affective and anxiety symptoms and personality traits

All individuals were assessed by a trained study clinician using the Structured Clinical Interview for DSM-5, Research Version (SCID-5-RV)^87^ to assess current and past psychiatric diagnostic status. Within 3 days of the neuroimaging assessment, a trained study clinician assessed the participant’s mood and anxiety symptom severity and personality traits, using well-validated scales and measures: the Hamilton Anxiety Rating Scale (HAMA)^65^ to assess anxiety, the Hamilton Rating Scale for Depression (HRSD)^66^ to assess depression, and the Young Mania Rating Scale (YMRS)^88^ to assess mania/hypomania (Table 1). The Neuroticism Extroversion Openness Five Factor Inventory (NEO PI-R)^64^ was used to assess personality traits during the initial clinical assessment. This analysis is part of a larger study that measured other symptoms and traits related to bipolar disorder (BD). The full list of measures is in the supplemental section.

### Exclusion criteria

Exclusion criteria were: history of head injury, neurological, pervasive developmental disorder or systemic medical disease; cognitive impairment (Mini-Mental State Examination^89^ score < 24, and premorbid NAART IQ^90^ estimate < 85; visual disturbance (< 20/40 Snellen visual acuity); left or mixed handedness (Annett criteria)^91^; alcohol/substance abuse/dependence (including nicotine); and/or illicit substance use (except cannabis as commonly used in young adults) over the last 3 months in distressed participants and lifetime in healthy controls (to avoid the influence of medications/substances on GM), determined by Structured Clinical Interview for DSM-5 (SCID)^87^ (and psychiatric records, if available). Urine tests on the scanning day excluded individuals with current illicit substance use (except cannabis); salivary alcohol tests excluded individuals who were intoxicated on the scanning day. Additional exclusion criteria were MRI screening exclusion criteria, and positive pregnancy test for female individuals or self-reporting of pregnancy; or taking any psychotropic medication for > 2 weeks for distressed/lifetime for controls. The full list of Exclusion criteria is in the supplemental section.

### MRI data acquisition and sMRI data analysis

Please see Supplemental Materials.

### Statistical analysis

1. To test the assumptions of linear regression Shapiro–Wilk normality tests were performed for depression, anxiety, and mania/hypomania residual score distributions.
2. To test Hypothesis 1, and given a large number of independent variables, a separate penalized regression elastic-net model was used for variable selection, using the GLMNET (4.1) package^92,93^ in R (version 4.0.3) for GM thickness. Elastic-net is a modified form of least squares regression that penalizes complex models with a regularization parameter (*λ*) and is sensitive to correlated variables^94,95^. The regularization parameter shrinks coefficients toward zero and eliminates unimportant terms^92^. tenfold cross-validation with an elastic net alpha = 0.5 identified the optimal penalty term (*λ*) that minimized mean cross-validated error, reduced the chances of overfitting, and enforced recommended sparsity in the solution^94^. λ of minimum mean cross-validated error was selected to identify the variables in a model with the least mean cross-validated error.
3. Multiple regression models (one each for depression, anxiety, and mania/hypomania as the dependent variable) in SPSS (version 27) were used to quantify effect sizes and the extent to which each identified variable from step 2, along with age, sex, and IQ, were associated with depression, anxiety, and mania/hypomania at a false discovery rate (FDR; *q* ≤ 0.05)^96^, to account for multiple comparisons. Given the high correlation among depression, anxiety, and mania/hypomania scores (Supplemental section, Table 1 all *r* > *0.7*) an additional analysis using a composite pathology score (rounded ratio) was performed to assess relationships among GM thickness and all three clinical measures. The composite pathology score was calculated as the sum of the three weighted scores X 100 and rounded to the nearest integer.
4. To test Hypothesis 2 mediation, the PROCESS macro bootstrapping algorithm was used^68^. Separate mediation models were tested for each significant GM variable (step 3) as independent variables (IVs) and depression, anxiety, and mania/hypomania scores as dependent variables (DVs). In each model, Neuroticism and its subfactors were operationalized as mediators to examine their potential influence on the statistically significant relationships identified between cortical thickness variables (step 3) and the severity of mood and anxiety symptoms^67^. False discovery rate correction (FDR; q ≤ 0.05) was used to account for multiple mediation tests. Bootstrapping (5000 iterations) was applied to calculate confidence intervals for mediating effects. To confirm the direction of the identified relationships, we also tested the alternative pathway with Neuroticism as IV, depression, anxiety, and mania/hypomania scores as DVs, and each GM variable as mediators.
5. Sensitivity analysis. Participants having current anxiety or depressive diagnoses were excluded, and regression analysis, with the appropriate family (see results 1 below), was performed on the remaining sample (*n* = 169).

## Results

The residual scores of depression, anxiety, and mania/hypomania did not meet regression assumptions (Shapiro–Wilk, *p* < 0.001), showed a positive skew, and had standard deviations larger than the means. We therefore used negative binomial models for all analyses^97^.
Results of Hypothesis 1 from the elastic-net-with-cross-validation models identified the non-zero variables listed in the Supplemental section, Table 2. Step 3 below shows the statistically significant results.

**Table 2:** Negative binomial regression analysis results of relationships between clinical outcome measures and predictors.

Hamilton depression rating scale–GM thickness model					95% Wald confidence interval for Exp (B)	
	*p*	q	B	Exp (B)	Lower	Upper
L inferior temporal	≤ *0.001*	**0.003**	− 5.64	0.004	0.00	0.07
L pars opercularis	*0.002*	**0.01**	4.87	130.22	5.57	3044.72
L transverse temporal	*0.02*	0.06	− 2.04	0.13	0.02	0.69
L isthmus cingulate	*0.03*	0.09	2.26	9.56	1.16	78.45
L temporal pole	*0.05*	0.10	− 1.09	0.33	0.11	0.98
L pericalcarine	0.18	0.34	2.16	8.67	0.36	209.17
L superior frontal	0.27	0.42	1.94	6.95	0.22	217.28
Sex	0.48	0.62	− 0.11	0.89	0.65	1.22
R postcentral	0.55	0.62	1.09	2.97	0.08	108.25
IQ	0.59	0.62	− 0.04	0.96	0.84	1.10
Age	0.620	0.62	− 0.03	0.97	0.85	1.10
Omnibus test (Likelihood Ratio Chi-Square = 1574,44 df = 11, *p* ≤ 0.001)						

Young mania rating scale–GM thickness model					95% Wald confidence interval for Exp (B)	
	*p*	q	B	Exp (B)	Lower	Upper
L pars opercularis	≤ *0.001*	** ≤ *0.001***	6.08	439.03	20.58	9364.40
L inferior temporal	≤ *0.001*	** ≤ *0.001***	− 6.47	0.002	5.65E−05	0.04
L isthmus cingulate	*0.03*	0.06	2.81	16.63	1.39	199.07
L temporal pole	0.07	0.12	− 1.31	0.27	0.07	1.10
Age	0.32	0.44	− 0.07	0.97	0.82	1.14
Sex	0.52	0.60	0.12	1.13	0.78	1.65
IQ	0.69	0.69	− 0.03	0.97	0.82	1.14
Omnibus test (Likelihood Ratio Chi-Square = 46.42, df = 7, *p* ≤ 0.001)						

Hamilton anxiety rating scale–GM thickness model					95% Wald confidence interval for Exp (B)	
	*p*	q	B	Exp (B)	Lower	Upper
L inferior temporal	≤ *0.001*	** ≤ 0.001**	− 6.77	0.00	6.23E−05	0.02
L pars opercularis	*0.002*	**0.01**	4.68	107.85	5.60	2075.19
L isthmus cingulate	*0.01*	**0.04**	2.84	17.05	1.93	150.76
L temporal pole	0.07	0.17	− 1.00	0.37	0.12	1.09
Sex	0.09	0.17	− 0.28	0.75	0.55	1.04
L parahippocampal	0.16	0.23	0.92	2.51	0.70	8.99
L pericalcarine	0.16	0.23	2.19	8.97	0.41	195.86
R postcentral	0.67	0.83	0.75	2.13	0.07	65.52
Age	0.86	0.86	− 0.01	0.99	0.86	1.13
IQ	0.86	0.86	− 0.01	0.99	0.86	1.13
Omnibus test (Likelihood Ratio Chi-Square = 1147.48, df = 10, *p* ≤ 0.001)						

CI, Confidence Interval; L, left; R, right; GM, Gray matter; q, False Discovery Rate (FDR). Benjamini–Hochberg FDR corrected adjusted *p*-value < .05. Exp (B) = odds ratio or a 1 unit change in predictor variable is an Exp (B) increase in the dependent variable (depression score).

Significant *p*-values in italics, FDR-corrected *q* in bold.
Regression analysis.
  3.i. **Depression** (Detailed statistical results, see Table 2 and Figure 1). Left pars opercularis thickness (*B* = 4.87, *p* = 0.002, *q* = 0.01) was positively related to depression. Left inferior temporal thickness (*B* = − 5.64, *p* ≤ 0.001, *q* = 0.003) was negatively related to depression.

**Figure 1:**
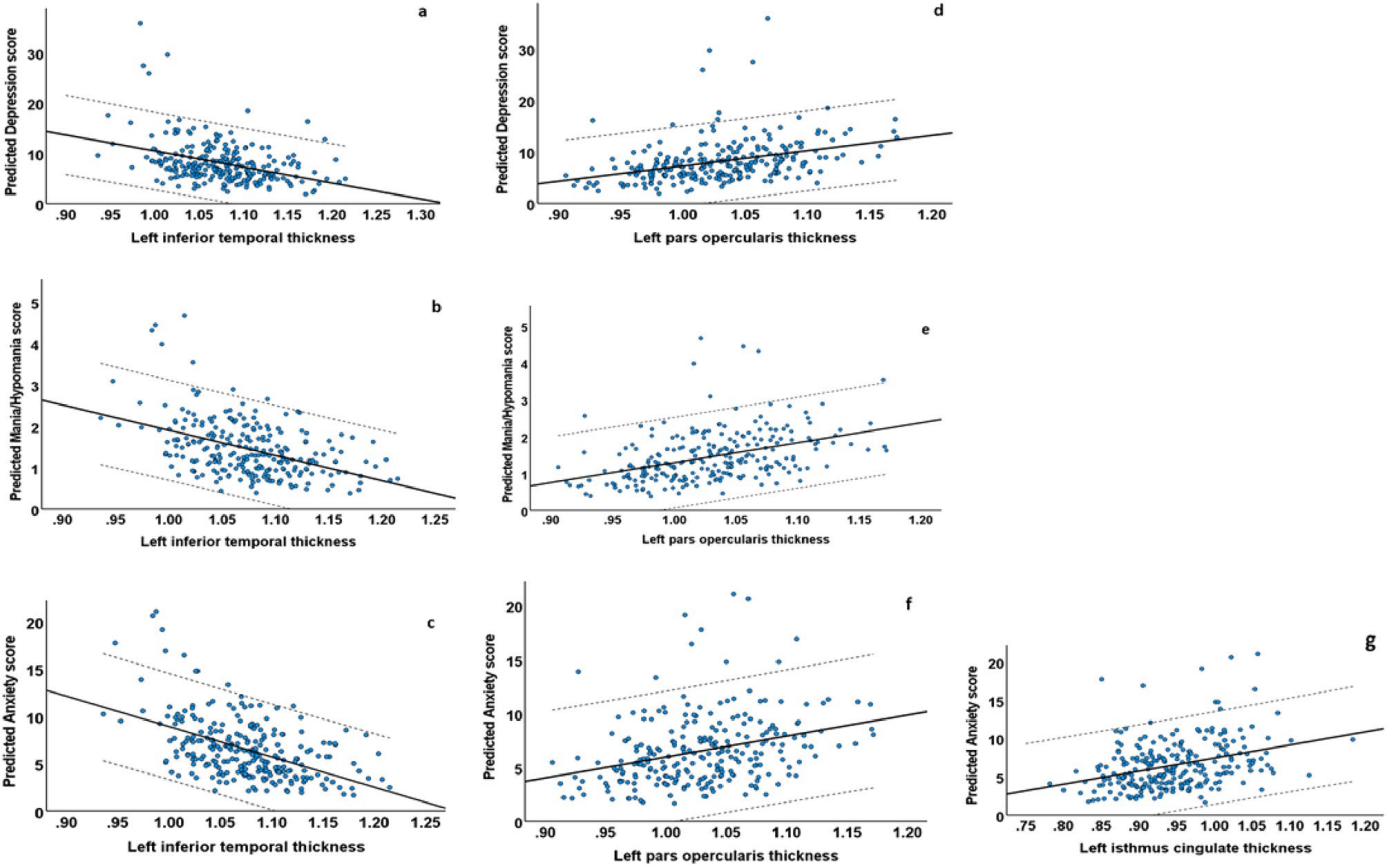
Scatterplots showing the relationships between GM thickness and mood and anxiety symptoms severity. (**a**) Predicted Hamilton Depression Scale and left inferior temporal thickness; (**b**) Predicted Young Mania Rating Scale and left inferior temporal thickness; (**c**) Predicted Hamilton Anxiety Scale and left inferior temporal thickness and left inferior temporal thickness; (**d**) Predicted Depression Scale and left pars opercularis thickness, (**e**) Predicted Young Mania Rating Scale and left pars opercularis thickness; (**f**) Predicted Hamilton Anxiety Scale and left pars opercularis thickness; (**g**) Predicted Hamilton Anxiety Scale and left isthmus cingulate thickness. Dashed lines represent 95% confidence intervals.
  3.ii. **Mania/hypomania** (Detailed statistical results, see Table 2 and Figure 1). Left pars opercularis thickness (*B* = 6.08, *p* ≤ 0.001, *q* ≤ 0.001) was positively related to mania/hypomania. Left inferior temporal thickness (*B* = − 6.47, *p* ≤ 0.001, *q* ≤ 0.001) was negatively related to mania/hypomania.
  3.iii. **Anxiety** (Detailed statistical results, see Table 2 and Figure 1). Left pars opercularis thickness (*B* = 4.68, *p* = 0.002, *q* = 0.009), and left isthmus cingulate thickness (*B* = 2.84, *p* = 0.01, *q* = 0.04) were positive related to anxiety. Left inferior temporal thickness (*B* = − 6.77, *p* ≤ 0.001, *q* ≤ 0.001) was negatively related to anxiety.
  3.iv. Composite pathology score (Detailed statistical results, see Supplemental section, Table 3). Left pars opercularis thickness (*B* = 5.49, *p* ≤ 0.001, *q* = 0.002) was positively related to the composite pathology score. Left inferior temporal thickness (*B* = − 6.13, *p* ≤ 0.001, *q* ≤ 0.001) was negatively related to the composite pathology score.
4. Mediation analysis.
  **Step I**. To test Hypothesis 2 mediation analysis for each of the seven significant relationships from Analysis 3 was performed. Neuroticism was thus used as a mediator in seven mediation models for the following relationships: depression and left inferior temporal thickness, left pars opercularis thickness; mania/hypomania and left inferior temporal thickness, left pars opercularis thickness; anxiety and left inferior temporal thickness, left pars opercularis thickness, and left isthmus cingulate thickness. The total effect reflects the relationship between GM and symptoms before the inclusion of the mediator; the direct effect reflects the relationship between GM and symptoms after the inclusion of the mediator; the indirect effect reflects the mediation effect of Neuroticism on the relationship between GM and symptoms. The “a” path reflects the relationship between GM and Neuroticism; the “b” path reflects the relationship between Neuroticism and symptoms. For detailed statistical results, see Table 3.

**Table 3:** Mediation analysis of effect of Neuroticism (NEO PI-R Five Factor Inventory) on relationships between GM metrics and depression, anxiety, and mania/hypomania symptoms.

	STEP I (factors)					
GM metric	Symptoms	Mediator	Type of mediation	Total effect *p*-value/FDR corrected q-value	Direct effect *p*-value	Indirect effect/BootLLCI/BootULCI
L inferior temporal thickness	Depression	Neuroticism	Full	0.005/0.02	0.08	− 17.45/− 32.95/− 2.75
L pars opercularis thickness	Depression	Neuroticism	None	0.04/0.05	0.08	
L pars opercularis thickness	Mania/hypomania	Neuroticism	None	0.007/0.02	0.01	
L inferior temporal thickness	Mania/hypomania	Neuroticism	Full	0.03/0.05	0.28	− 2.91/− 5.47/− 0.43
L inferior temporal thickness	Anxiety	Neuroticism	Partial	0.001/0.007	0.02	− 14.52/− 27.09/− 2.27
L isthmus cingulate thickness	Anxiety	Neuroticism	None	0.05/0.06	0.09	
L pars opercularis thickness	Anxiety	Neuroticism	None	0.07/0.07	0.19	
	STEP II (subfactors)					
GM metric	Symptoms	Mediator	Type of mediation	Total effect *p*-value/FDR corrected q-value	Direct effect *p*-value	Indirect effect/BootLLCI/BootULCI
L inferior temporal thickness	Depression	N6	Full	0.005/0.008	0.08	− 6.36/− 12.85/− 1.57
L inferior temporal thickness	Mania/hypomania	N2	Full	0.03/0.03	0.44	− 1.19/− 2.66/− 0.18
L inferior temporal thickness	Anxiety		None	0.001/0.004	0.02	

	STEP II (subfactors)					
GM metric	Symptoms	Mediator	Type of mediation	Total effect *p*-value/FDR corrected q-value	Direct effect *p*-value	Indirect effect/BootLLCI/BootULCI
L inferior temporal thickness	Depression	N6	Full	0.005/0.008	0.08	− 6.36/− 12.85/− 1.57
L inferior temporal thickness	Mania/hypomania	N2	Full	0.03/0.03	0.44	− 1.19/− 2.66/− 0.18
L inferior temporal thickness	Anxiety		None	0.001/0.004	0.02	

L, left; R, right; GM, Gray matter; BootLLCI/BootULCI, Bootstrap Confidence Intervals; N2, Anger/hostility; NEO, Neuroticism subfactor; N6, Vulnerability; NEO, Neuroticism subfactor.

**Figure 2:**
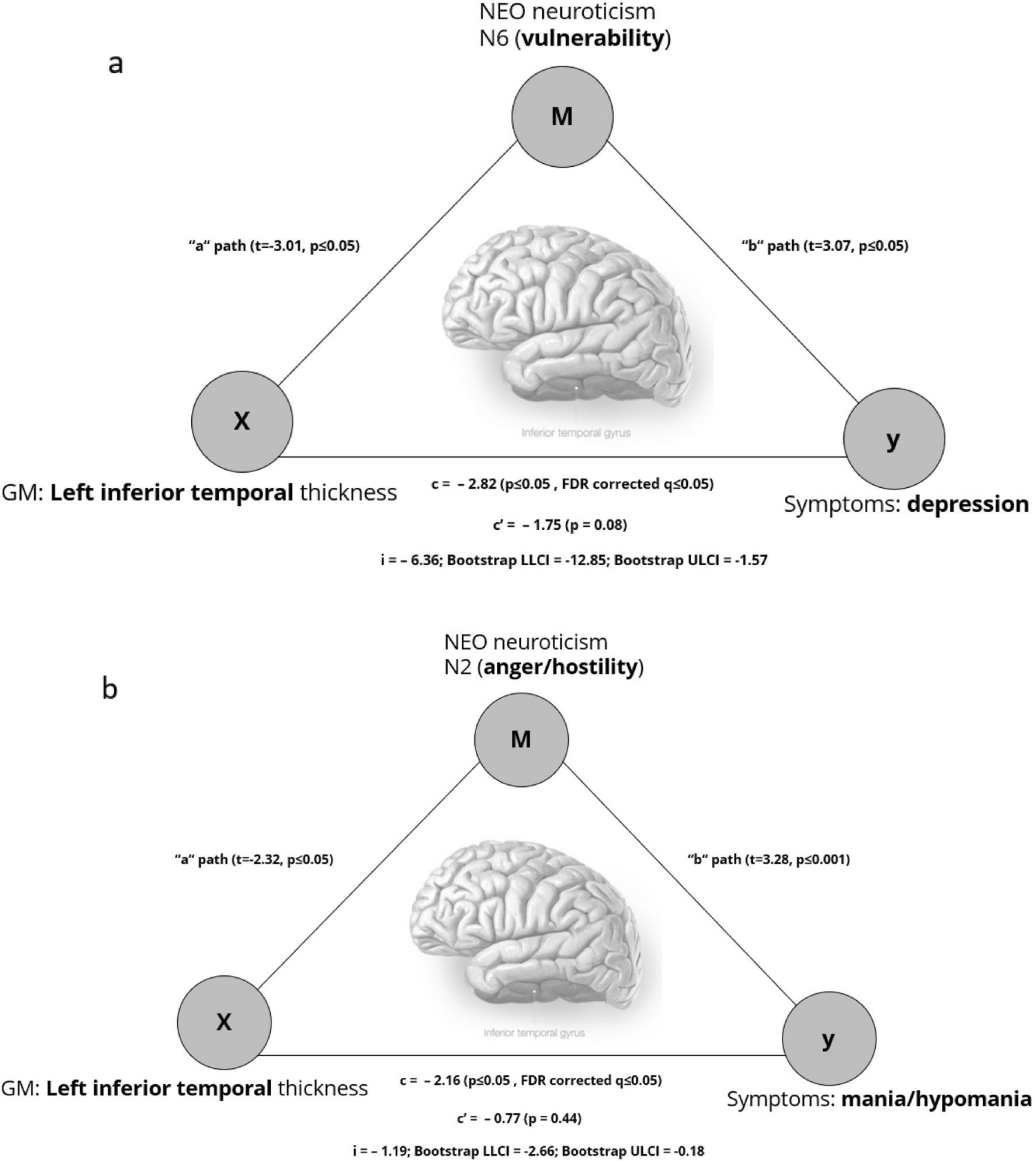
(**a**) Neuroticism subfactor N6 (vulnerabily) mediates the relationship between Left inferior temporal thickness and severity of depression, (**b**) Neuroticism subfactor N2 (anger/hostility) mediates the relationship between Left inferior temporal thickness and severity of mania/hypomania. X–GM thickness, M–mediator, Y–symptoms severity. (“a” path—the relationship between GM and neuroticism subfactor, “b” path—the relationship between neuroticism subfactor and symptoms, c—the total effect of the relationship between GM and symptoms, c’—the direct effect of the relationship between GM and symptoms, i—the indirect effect, CL—Bootstrap confidence intervals).

    4.i. **Depression**. Neuroticism fully mediated the negative relationship between left inferior temporal thickness and depression (*total effect: t* = − 2.82, *p* ≤ 0.05; *q* ≤ 0.05; *direct effect: t* = − 1.73, *p* = 0.085; *indirect effect* = − 17.45, *BootLLCI* = − 32.95, *BootULCI* = − 2.75; “*a*” *path: t* = − 2.22, *p* ≤ 0.05; “*b*”* path: t* = 18.73, *p* ≤ 0.001).
    4.ii. **Mania/hypomania**. Neuroticism fully mediated the negative relationship between left inferior temporal thickness and mania/hypomania (*total effect: t* = − 2.16, *p* ≤ 0.05; *q* ≤ 0.05; *direct effect: t* = − 1.09, *p* = 0.276; *indirect effect* = − 2.91, *BootLLCI* = − 5.47, *BootULCI* = − 0.43; “*a*” *path: t* = − 2.22, *p* ≤ 0.05; “*b*”* path: t* = 10.81, *p* ≤ 0.001).
    4.iii. **Anxiety**. Neuroticism partially mediated the negative relationship between left inferior temporal thickness and anxiety (*total effect: t* = − 3.24, *p* ≤ 0.001; *q* ≤ 0.05;* direct effect: t* = − 2.36,* p* ≤ 0.05; *indirect effect* = − 14.52,* BootLLCI* = − 27.09, *BootULCI* = − 2.27; “*a*”* path: t* = − 2.22,* p* ≤ 0.05; “*b*”* path: t* = 17.63, *p* ≤ 0.001).
  **Step II** (Figure 2). One mediation analysis of neuroticism subfactors (N1 (anxiety), N2 (anger/hostility), N3 (depression), N4 (self-consciousness), N5 (impulsiveness), and N6(vulnerability) was performed for each DV (depression, mania, anxiety) and the left inferior temporal thickness that showed full or partial mediation in Analysis 5 Step I and survived FDR correction. For detailed statistical results, see Table 3.

    4.iv. **Depression.** The NEO PI-R N6 subfactor (vulnerability) fully mediated the negative relationship between left inferior temporal thickness and depression (*total effect: t* = − 2.82, *p* ≤ 0.05; *q* ≤ 0.05; *direct effect: t* = − 1.75, *p* = 0.08; *indirect effect: t* = − 6.36, *BootLLCI* = − 12.85, *BootULCI* = − 1.57; “*a*” *path: t* = − 3.01, *p* ≤ 0.05; “*b*” *path: t* = 3.07, *p* ≤ 0.05).
    4.v. **Mania/hypomania**. The NEO PI-R N2 subfactor (anger/hostility) fully mediated the negative relationship between left inferior temporal thickness and mania/hypomania (*total effect: t* = − 2.16, *p* ≤ 0.05; *q* ≤ 0.05; *direct effect: t* = − 0.77, *p* = 0.44; *indirect effect* = − 1.19, *BootLLCI* = − 2.66, *BootULCI* = − 0.18; “*a*”* path: t* = − 2.32, *p* ≤ 0.05; “*b*”* path: t* = 3.28, *p* ≤ 0.001).
    4.vi. **Anxiety**. No Neuroticism subfactor showed any mediation effect on negative relationships between left inferior temporal thickness and anxiety.
    4.vii. **Alternative pathways**. No mediation effect of left inferior temporal thickness as a mediator was found on the positive relationship between the NEO PI-R N6 subfactor (vulnerability) and depression (*total effect: t* = 16.55, *p* ≤ 0.001; *direct effect: t* = 16.07, *p* ≤ 0.001; *indirect effect* = 0.01, *BootLLCI* = − 0.01, *BootULCI* = 0.04), and on the positive relationships between the NEO PI-R N2 subfactor (anger/hostility) and mania/hypomania (*total effect: t* = 8.77, *p* ≤ 0.001; *direct effect: t* = 8.51, *p* ≤ 0.001; *indirect effect* = 0.003, *BootLLCI* = − 0.001, *BootULCI* = 0.01).
**Sensitivity analysis**. The analysis on the sample excluding participants with a current depression or anxiety diagnosis (*n* = 169) confirmed our main findings: left inferior temporal thickness was negatively related to anxiety (*B* = − 4.92, *p* = 0.01, *q* = 0.04), depression (*B* = − 6.12, *p* = 0.005, *q* = 0.03), and mania/hypomania (*B* = − 6.53, *p* = 0.007, *q* = 0.01); left pars opercularis thickness was positively related to anxiety (*B* = 7.21, *p* ≤ 0.001, *q* = 0.009), depression (*B* = − 5.71, *p* = 0.01,* q* = 0.04), and mania/hypomania (*B* = − 9.45, *p* ≤ 0.001, *q* ≤ 0.001) (Supplemental section, Table 5).

## Discussion

Given that the role of GM cortical thickness in psychopathology remains unclear, we aimed to identify neural markers, associated with mood and anxiety symptoms in a large sample of young adults recruited across a range of subsyndromal to syndromal levels of these symptoms. Additionally, we explored the extent to which neuroticism mediated these relationships. We demonstrated common and distinct patterns of relationships between GM thickness in the left inferior temporal gyrus, the left pars opercularis, and the left isthmus cingulate, in relation to subsyndromal-syndromal symptoms. Specifically, we showed that cortical thickness in the left inferior temporal gyrus was negatively related to depression, anxiety, mania/hypomania, and composite pathology score, while cortical thickness in the left pars opercularis was positively related to these measures. Thickness in the left isthmus cingulate cortex was positively related to anxiety. The results also indicate that some of these relationships were mediated by neuroticism, where specific neuroticism subfactors explained the significant relationships between left inferior temporal gyrus cortical thickness and both mania/hypomania and depression. Specifically, the NEO subfactor anger/hostility mediated the negative relationship between left inferior temporal gyrus thickness and mania/hypomania; while the NEO subfactor vulnerability to stress mediated the relationship between left inferior temporal gyrus thickness and depression. The specificity of the directions of these pathways were supported by showing that cortical thickness measures did not mediate relationships between the above Neuroticism subfactors and mania/hypomania and depression. To our knowledge, this is the first study to examine tripartite relationships among cortical thickness, neuroticism subfactors, and mood and anxiety disorder risk.

Partially consistent with our first hypothesis, we observed negative relationships between left inferior temporal cortical thickness and the severity of anxiety, depression, mania/hypomania symptoms, aligning with previous studies that reported reduced inferior temporal gyrus thickness in adults with mood disorders^24,29,30,35,36^, and childhood abuse exposure and severity in adolescents^98^. Additionally, our composite pathology score supported this negative relationship with left inferior temporal cortical thickness. This region is known as a key part of the visual pathway implicated in object, face, and scene perception^99^. Functional connectivity studies have shown that this region is related to text-based memory^100^, and atrophy in this part of cortex is related to semantic dementia^101^. Taken together, these findings may point to cognitive deficits observed in both mood and anxiety^102–105^, underscoring the transdiagnostic relevance of this region in the susceptibility to mood and anxiety disorders.

We observed positive relationships between left pars opercularis thickness and severity of anxiety, depression, and mania/hypomania symptoms. Moreover, our analysis using the composite pathology score showed this same positive relationship with pars left pars opercularis, a part of ventrolateral prefrontal cortex, is involved in language production and comprehension^106^, and supports attention to unexpected stimuli^107–110^. These findings only partially support our hypotheses and parallel previous reports of a greater GM thickness in left pars opercularis in adults with depression^34,48^; however, other studies have shown lower cortical thickness in vlPFC in adults with BD^31,36,37^ and related to the number of (hypo)manic episodes^111^. Given that the vlPFC supports attention to unexpected and especially salient (e.g., negative emotional) stimuli^107–110^, and voluntary emotion regulation^112^, greater cortical thickness in this region might underlie greater attention to unexpected and negative emotional events in at-risk young adults. The combination of reduced inferior temporal gyrus thickness and associated cognitive deficits, and greater vlPFC cortical thickness associated with greater attention to unexpected and negative events, might thus result in greater mood and anxiety symptom severity.

Additionally, we observed anxiety-specific cortical thickening in the left isthmus cingulate cortex. This region supports internally directed thought^113–115^, and is involved in imagination, formation and consolidation of episodic memory^116–119^, and self-relevance assessment^120^. Given that we have not found an association between cortical thickness in this region and depression or mania/hypomania symptoms, perhaps thickening of the left isthmus cingulate cortex may suggest a link between internal thoughts and self-related memories and assessments, and development of anxiety symptoms. Thickness in this region may also help to differentiate the risk for anxiety disorders from the risk for mood disorders.

In support of our second hypothesis, relationships between GM cortical thickness and mood and anxiety symptoms severity were mediated by neuroticism, and by two neuroticism subfactors in particular—anger/hostility, and vulnerability. The anger/hostility subfactor mediated the relationship between left inferior temporal gyrus cortical thickness and mania/hypomania, where the relationship between lower inferior temporal gyrus cortical thickness and greater mania/hypomania severity was explained by higher levels of anger/hostility. Greater levels of anger have been reported in individuals with BD^121–123^. Our findings might thus reflect a neural mechanism linking neuroticism-related anger and predisposition to mania/hypomania. In addition, the vulnerability to stress subfactor mediated the relationship between left inferior temporal gyrus thickness and depression, where the relationship between lower inferior temporal gyrus cortical thickness and greater depression was explained by greater vulnerability, in support of the established role of stress in depression^124–126^. These mediation findings together suggest that neuroticism along with decreased left inferior temporal cortical thickness plays an important role in young adults at risk to mood, but not anxiety disorders. The fact that the mediation effects were shown to the vulnerability and not the depression subfactor of neuroticism might reflect differences in the nature of the HRSD and NEO PI-R depression subfactor as discussed above. Taken together, these findings may contribute to earlier diagnosis and treatment of mood disorders.

A potential limitation of the study was the heterogenous sample, with some participants having anxiety and depressive disorders. Sensitivity analyses showed, however, that in participants without depressive or anxiety disorders, our principal findings remained consistent: a negative relationship was observed between left inferior temporal thickness and all three measures of symptom severity, while a positive relationship was identified with left pars opercularis thickness. Although our study included a large sample of young adults, replication of our findings in future studies is needed. Longitudinal studies can provide more precise inferences about the potential directionality and consistency of these relationships over time. It will also be beneficial to test these relationships in other age groups along with sex differences.

To our knowledge, the present study of a large sample of unmedicated young adults recruited across a broad range of subsyndromal to syndromal mood and anxiety symptoms measured with clinically valid and sensitive assessments, is the first to explore tripartite relationships among cortical GM thickness, neuroticism and its subfactors, and mood and anxiety symptoms severity. Given that neuroticism is closely related to mood and anxiety symptoms, examining the interrelationships among cortical thickness, neuroticism and mood and anxiety symptoms enriches the potential for identifying markers conferring risk for mood and anxiety disorders and targets for personalized intervention strategies for these disorders.

### Supplementary Information

Supplementary Information.

## Data Availability

The datasets used and/or analyzed during the current study available from the corresponding author on reasonable request.
