# Supplementary material for: Identifying tripartite relationship among cortical thickness, neuroticism, and mood and anxiety disorders
Source: Sci Rep. 2024 Apr 11;14:8449. doi: 10.1038/s41598-024-59108-1 (PMC11006921; doi:10.1038/s41598-024-59108-1)
Supplement: Supplementary file 1 — Supplementary Information. [file 41598_2024_59108_MOESM1_ESM.docx]

**Supplemental Methods and results**

**Clinical and behavioral assessments in a broader study**

Snaith-Hamilton Pleasure Scale (SHAPS) ^1^, The Brief Psychiatric Rating Scale Expanded Version (BPRS-E; original ^2^ expanded ^3^, Kessler Psychological Distress Scale (K10) ^4,5^, Positive And Negative Affect Scale (PANAS-I) ^6^, Trauma History Questionnaire (THQ) ^7^, Mood and Anxiety Symptom Questionnaire (MASQ) ^8^, Monetary Choice Questionnaire (MCQ), Quality of Life Enjoyment and Satisfaction Questionnaire-Short Form (Q-LES-Q-SF) ^9^, Spielberger State & Trait Anxiety Inventory (STAIY-1) ^10^. On the day of the scan were administered the Structured Clinical Interview for DSM-5 (SCID) ^11^, Barratt Simplified Measure of Social Status (BSMSS), North American National Adult Reading Test NAART ^12^, The Mini-Mental Status Examination, MMSE ^13^, The Longitudinal Interval Follow-up Evaluation (LIFE) Adaptation for Longitudinal Outcome (Bauer, personal communication) ^14–16^, The Longitudinal Interval Follow-Up Evaluation-Range of Impaired Functioning (LIFE-RIFT) ^17^, Kessler Psychological Distress Scale (K10) ^4,5^, Sensation Seeking Scale (SSS)^18^, Barratt Impulsiveness Scale (BIS-11)^19^, Behavioral Inhibition and Activation Scales (BIS/BAS) ^20^, Impulsive Behavior Scale (UPPS-P), MOODS Self-Report (MOODS-SR) ^21,22^. Participants were clinically followed for up to 24 months after the baseline scan.

**Exclusion criteria assessments**

Premorbid NART IQ estimate ^12^, Behavioral Handedness Index (BHI) ^23^, Snellen Visual Acuity Testing, Cigarette/Nicotine Dependence Scale (CDS), and salivary alcohol and urine drug screening, urine pregnancy test (as appropriate) were assessed.

**Exclusion criteria**

Exclusion criteria were: history of head injury, neurological, pervasive developmental disorder or systemic medical disease; cognitive impairment (Mini-Mental State Examination ^13^ score<24, and premorbid NAART IQ ^12^ estimate<85; visual disturbance(<20/40 Snellen visual acuity); left or mixed handedness(Annett criteria)^23^; participating in another study that used a card-guessing task; alcohol/substance abuse/dependence (including nicotine) and/or illicit substance use (except cannabis as commonly used in young adults) over the last 3 months in distressed participants and lifetime in healthy controls, determined by Structured Clinical Interview for DSM-5 (SCID) ^11^ (and psychiatric records, if available). Urine tests on the scanning day excluded individuals with current illicit substance use (except cannabis); salivary alcohol tests excluded individuals who were intoxicated on the scanning day. Additional exclusion criteria were MRI screening exclusion criteria, and positive pregnancy test for female individuals or self-reporting of pregnancy; or taking any psychotropic medication for >2 weeks for distressed/lifetime for controls. Previous use of psychotropic medications was permitted after 3 months of stopping medications in the distressed.

***MRI data acquisition***

Structural 3D axial MPRAGE images were acquired on two scanners at the MRI Research Center (MRRC) in Pittsburgh 1. 3.0 Tesla Siemens Trio 2 MRI scanner (Siemens Medical Solutions, Erlangen, Germany) (128 participants) (TR·TE = 2200/3.29 ms; flip angle=9°, FOV = 256x192mm; 1 mm isotropic voxels; 192 continuous slices), and 2. 3.0 Tesla Siemens Prisma scanner (124 participants) (TR·TE = 1520/3.17 ms; flip angle 8°; FOV = 256 × 256 mm; 1 mm isotropic voxels; 176 continuous slices).

***sMRI data analysis***

Structural T1 weighted images were processed, and regional cortical thickness was estimated using Freesurfer (Version 6.0, <http://surfer.nmr.mgh.harvard.edu/>) an automated surface reconstruction and segmentation/parcellation previously described procedures ^24–26^. GM thickness measures, according to the Desikan-Killiany-Tourville atlas ^27^ were exported to SPSS (version 27, IBM Corp. Released 2020. IBM SPSS Statistics for Windows, Version 27.0. Armonk, NY: IBM Corp) for use in statistical packages for analyses described below. Given that the data were collected on two different MRI scanners(Siemens Prisma and Siemens Trio), data were harmonized using ComBat software^28^, with the recommended use of age and gender as covariates, for further analysis. GM thickness was normalized to control for brain size by dividing individual thickness by mean cortical thickness.

**Sensitivity analysis methods and results**

This analysis was performed in a subsample of participants without a current diagnosis of an anxiety or depressive disorder (*n=169, age 21.8 (SD=1.9*), 114 females). Negative binomial regression was performed in SPSS. The analysis confirmed our main findings - left inferior temporal thickness was negatively related to anxiety (*B=-4.92, p=0.01*), depression (*B=-6.12, p=0.005*), and mania (*B=-6.53, p=0.007*); left pars opercularis thickness was positively related to anxiety (*B=7.21, p≤0.001*), depression (*B=-5.71, p=0.01*), and mania (*B=-9.45, p≤0.001*) (SM table 4).

*Supplemental Table 1. Spearman Correlations between Hamilton Anxiety scale, Hamilton Rating Scale for Depression, and Young Mania Rating Scale.*

|  |  |  | HAMA | HRSD | YMRS | Neuroticism |
| --- | --- | --- | --- | --- | --- | --- |
| Spearman's rho | HAMA | Correlation Coefficient | 1.000 | .958** | .796** | .778** |
|  |  | Sig. (2-tailed) | . | ≤0.001 | ≤0.001 | ≤0.001 |
|  |  | N | 252 | 252 | 252 | 252 |
|  | HRSD | Correlation Coefficient | .958** | 1.000 | .793** | .789** |
|  |  | Sig. (2-tailed) | .000 | . | .000 | .000 |
|  |  | N | 252 | 252 | 252 | 252 |
|  | YMRS | Correlation Coefficient | .796** | .793** | 1.000 | .659** |
|  |  | Sig. (2-tailed) | ≤0.001 | ≤0.001 | . | ≤0.001 |
|  |  | N | 252 | 252 | 252 | 252 |

**. Correlation is significant at the 0.01 level (2-tailed).

*Supplemental Table 2.* *Coefficients of cross validated non-zero GM variables from the elastic net regression analysis.* ***Abbreviations: L = left, R = right, GM=Gray matter***

| ***Anxiety*** | | ***Depression*** | | ***Mania***/hypomania | |
| --- | --- | --- | --- | --- | --- |
| ***GM thickness model*** | | | | | |
| ***Anxiety*** | | ***Depression*** | | ***Mania***/hypomania | |
| L inferior temporal | -2.39 | L inferior temporal | -1.93 | L inferior temporal | -1.10 |
| L pars opercularis | 2.29 | L pars opercularis | 2.43 | L pars opercularis | 2.24 |
| L isthmus cingulate | 1.53 | L isthmus cingulate | 1.08 | L isthmus cingulate | 0.51 |
| L pericalcarine | 0.23 | L pericalcarine | 0.31 | L temporal pole | -0.22 |
| L temporal pole | -0.13 | L temporal pole | -0.20 |  |  |
| R postcentral | 0.67 | R postcentral | 0.27 |  |  |
| L parahippocampal | 0.08 | L transverse temporal | -0.36 |  |  |
|  |  | L superior frontal | 0.71 |  |  |

*Supplemental Table 3. Negative binomial regression analysis of regions related to rounded ratio of scores from Anxiety, Depression, and Mania scales. Abbreviations: Confidence Interval (CI), L=left, R=right, GM=Gray matter, q= False Discovery Rate (FDR). Benjamini-Hochberg FDR corrected adjusted p-value <.05. Exp(B) = odds ratio or a 1-unit change in predictor variable is an Exp(B) increase in the dependent variable (rounded ratio score).*

| *Composite pathology score – GM* ***thickness*** *model* | | | | | 95% Wald Confidence Interval for Exp(B) | |
| --- | --- | --- | --- | --- | --- | --- |
|  | p | q | B | Exp(B) | Lower | Upper |
| L inferior temporal | *≤0.001* | **≤0.001** | -6.13 | 0.002 | 0.00 | 0.04 |
| L pars opercularis | *≤0.001* | **0.002** | 5.49 | 243.02 | 11.74 | 5029.93 |
| L isthmus cingulate | *0.02* | 0.05 | 2.54 | 12.70 | 1.60 | 100.60 |
| L transverse temporal | *0.02* | 0.05 | -1.91 | 0.15 | 0.03 | 0.72 |
| L temporal pole | *0.03* | 0.07 | -1.14 | 0.32 | 0.11 | 0.89 |
| L pericalcarine | 0.08 | 0.15 | 2.83 | 16.97 | 0.74 | 390.18 |
| L parahippocampal | 0.08 | 0.15 | 1.07 | 2.92 | 0.86 | 9.88 |
| Gender | 0.35 | 0.53 | -0.14 | 0.87 | 0.64 | 1.17 |
| L superior frontal | 0.43 | 0.58 | 1.33 | 3.79 | 0.13 | 106.31 |
| R postcentral | 0.57 | 0.61 | 0.98 | 2.68 | 0.08 | 84.30 |
| Age | 0.58 | 0.61 | -0.04 | 0.96 | 0.85 | 1.10 |
| IQ | 0.61 | 0.61 | -0.03 | 0.99 | 0.85 | 1.10 |
| Omnibus test (Likelihood Ratio Chi-Square=8229.96, df=12, p≤ 0.001) | | | | | | |

*Supplemental Table 4. Sensitivity analysis. Negative binomial regression analysis results of relationships between clinical outcome measures and predictors in participants without a current depressive and/or anxiety disorder (n=146).* *Abbreviations: Confidence Interval (CI), L=left, R=right, GM=Gray matter****,*** *q False Discovery Rate (FDR). Benjamini-Hochberg FDR corrected adjusted p-value <.05. Exp(B) = odds ratio or a 1-unit change in predictor variable is an Exp(B) increase in the dependent variable.*

| *Hamilton* ***Depression*** *Rating Scale – GM* ***thickness*** *model* | | | | | 95% Wald Confidence Interval for Exp(B) | |
| --- | --- | --- | --- | --- | --- | --- |
|  | p | q | B | Exp(B) | Lower | Upper |
| L inferior temporal | *0.005* | **0.030** | -6.12 | 0.002 | 2.921E-05 | 0.16 |
| L pars opercularis | *0.010* | **0.039** | 5.72 | 301.21 | 3.79 | 23955.22 |
| L transverse temporal | 0.051 | 0.113 | -2.24 | 0.11 | 0.01 | 1.015 |
| L isthmus cingulate | 0.263 | 0.383 | 1.62 | 5.04 | 0.30 | 86.01 |
| L temporal pole | 0.004 | 0.030 | -2.15 | 0.12 | 0.03 | 0.51 |
| L pericalcarine | 0.504 | 0.616 | 1.38 | 3.97 | 0.07 | 226.93 |
| L superior frontal | 0.049 | 0.113 | 4.59 | 98.13 | 1.03 | 9304.71 |
| Gender | 0.088 | 0.162 | 0.34 | 1.41 | 0.95 | 2.10 |
| R postcentral | 0.759 | 0.759 | -0.72 | 0.49 | 0.005 | 48.26 |
| IQ | 0.278 | 0.383 | -0.09 | 0.91 | 0.78 | 1.07 |
| Age | 0.666 | 0.733 | 0.04 | 1.04 | 0.87 | 1.25 |
| Omnibus test (Likelihood Ratio Chi-Square= 314.75, df=11, p≤ 0.001) | | | | | | |
| *Young* ***Mania*** *Rating Scale – GM* ***thickness*** *model* | | | | | 95% Wald Confidence Interval for Exp(B) | |
|  | p | q | B | Exp(B) | Lower | Upper |
| L pars opercularis | *≤0.001* | ***≤0.001*** | 9.45 | 12777.81 | 110.92 | 1471961.52 |
| L inferior temporal | *0.007* | **0.012** | -6.53 | *≤0.001* | 1.254E-05 | 0.17 |
| L isthmus cingulate | 0.671 | 0.671 | 0.81 | 2.26 | 0.05 | 96.86 |
| L temporal pole | 0.007 | 0.012 | -3.05 | 0.05 | 0.005 | 0.43 |
| Age | 0.337 | 0.471 | -0.13 | 0.88 | 0.68 | 1.14 |
| Gender | 0.002136 | 0.007 | 0.81 | 2.25 | 1.34 | 3.79 |
| IQ | 0.415 | 0.485 | -0.09 | 0.92 | 0.74 | 1.13 |
| Omnibus test (Likelihood Ratio Chi-Square=32.81, df=7, p≤0.001) | | | | | | |
| *Hamilton* ***Anxiety*** *Rating Scale – GM* ***thickness*** *model* | | | | | 95% Wald Confidence Interval for Exp(B) | |
|  | p | q | B | Exp(B) | Lower | Upper |
| L inferior temporal | *0.01* | **0.04** | -4.92 | 0.007 | 0 | 0.36 |
| L pars opercularis | *0.001* | **0.01** | 7.21 | 1358.91 | 18.77 | 98383.89 |
| L isthmus cingulate | 0.24 | 0.59 | 1.77 | 5.85 | 0.31 | 108.60 |
| L temporal pole | 0.009 | 0.04 | -2.06 | 0.13 | 0.03 | 0.60 |
| Gender | 0.46 | 0.66 | 0.15 | 1.17 | 0.77 | 1.75 |
| L parahippocampal | 0.77 | 0.77 | 0.27 | 1.31 | 0.22 | 7.73 |
| L pericalcarine | 0.37 | 0.65 | 1.79 | 5.98 | 0.12 | 288.70 |
| R postcentral | 0.39 | 0.65 | -1.93 | 0.14 | 0 | 12.05 |
| Age | 0.77 | 0.77 | 0.03 | 1.03 | 0.85 | 1.25 |
| IQ | 0.75 | 0.77 | -0.03 | 0.97 | 0.82 | 1.15 |
| Omnibus test (Likelihood Ratio Chi-Square=*163.45,* df=10, p*≤0.001*) | | | | | | |

*Supplemental* *Table 5.* **Range of GM values in the regions having significant relationships with anxiety, depression, and mania.**

|  | Left pars opercularis thickness | Left Inferior temporal thickness | Left isthmus cingulate thickness |
| --- | --- | --- | --- |
| Mean | 1.029 | 1.075 | 0.945 |
| Std. Deviation | 0.052 | 0.050 | 0.064 |
| Variance | 0.003 | 0.003 | 0.004 |
| Range | 0.266 | 0.279 | 0.402 |

**References**

1. Snaith, R. P. *et al.* A scale for the assessment of hedonic tone the Snaith-Hamilton Pleasure Scale. *Br J Psychiatry* **167**, 99–103 (1995).

2. Overall, J. & Gorham, D. THE BRIEF PSYCHIATRIC RATING SCALE. *Psychological Reports.* **10**, 799–812 (1962).

3. Ventura, J. L., Nuechterlein KH, Liberman RL, Green MF & Shaner A. The Brief Psychiatric Rating Scale (version 4.0) factorial structure and its sensitivity in the treatment of outpatients with unipolar depression. *Psychiatry Res* **210**, 626–633 (2013).

4. Kessler, R. C. *et al.* Short screening scales to monitor population prevalences and trends in non-specific psychological distress. *Psychol Med* **32**, 959–976 (2002).

5. Andrews, G. & Slade, T. Interpreting scores on the Kessler Psychological Distress Scale (K10). *Aust N Z J Public Health* **25**, 494–497 (2001).

6. Watson, D., Clark, L. A. & Tellegen, A. Development and validation of brief measures of positive and negative affect: the PANAS scales. *J Pers Soc Psychol* **54**, 1063–1070 (1988).

7. Hooper, L. M., Stockton, P., Krupnick, J. L. & Green, B. L. Development, Use, and Psychometric Properties of the Trauma History Questionnaire. *Journal of Loss and Trauma* **16**, 258–283 (2011).

8. Clark, L. A. & Watson, D. Tripartite model of anxiety and depression: psychometric evidence and taxonomic implications. *J Abnorm Psychol* **100**, 316–336 (1991).

9. Endicott, J., Nee, J., Harrison, W. & Blumenthal, R. Quality of Life Enjoyment and Satisfaction Questionnaire: a new measure. *Psychopharmacol Bull* **29**, 321–326 (1993).

10. Spielberger, C. D. State-Trait Anxiety Inventory. in *The Corsini Encyclopedia of Psychology* (John Wiley & Sons, Ltd, 2010). doi:10.1002/9780470479216.corpsy0943.

11. First, M., Williams JBW, Karg RS & Spitzer RL. *Structured Clinical Interview for DSM-5—Research Version (SCID-5 for DSM-5, Research Version; SCID-5-RV).* (2015).

12. Blair, J. R. & Spreen, O. Predicting premorbid IQ: A revision of the national adult reading test. *Clinical Neuropsychologist* **3**, 129–136 (1989).

13. Folstein, M. F., Folstein, S. E. & McHugh, P. R. ‘Mini-mental state’. A practical method for grading the cognitive state of patients for the clinician. *J Psychiatr Res* **12**, 189–198 (1975).

14. Judd, L. L. *et al.* A prospective investigation of the natural history of the long-term weekly symptomatic status of bipolar II disorder. *Arch Gen Psychiatry* **60**, 261–269 (2003).

15. Keller, M. B. *et al.* The Longitudinal Interval Follow-up Evaluation. A comprehensive method for assessing outcome in prospective longitudinal studies. *Arch Gen Psychiatry* **44**, 540–548 (1987).

16. Simon, G. E. *et al.* Randomized trial of a population-based care program for people with bipolar disorder. *Psychol Med* **35**, 13–24 (2005).

17. Leon, A. C. *et al.* The Range of Impaired Functioning Tool (LIFE-RIFT): a brief measure of functional impairment. *Psychol Med* **29**, 869–878 (1999).

18. Zuckerman, M. The sensation seeking scale V (SSS-V): Still reliable and valid. *Personality and Individual Differences* **43**, 1303–1305 (2007).

19. Patton, J. H., Stanford, M. S. & Barratt, E. S. Factor structure of the Barratt impulsiveness scale. *J Clin Psychol* **51**, 768–774 (1995).

20. Carver, C. S. & White, T. L. Behavioral inhibition, behavioral activation, and affective responses to impending reward and punishment: The BIS/BAS Scales. *Journal of Personality and Social Psychology* **67**, 319–333 (1994).

21. Miniati, M. *et al.* Sensitivity to Change and Predictive Validity of the MOODS-SR Questionnaire, Last-Month Version. *Psychother Psychosom* **78**, 116–124 (2009).

22. Dell’Osso, L. *et al.* Measuring mood spectrum: comparison of interview (SCI-MOODS) and self-report (MOODS-SR) instruments. *Compr Psychiatry* **43**, 69–73 (2002).

23. Annett, M. A classification of hand preference by association analysis. *Br J Psychol* **61**, 303–321 (1970).

24. Dale, A. M., Fischl, B. & Sereno, M. I. Cortical surface-based analysis. I. Segmentation and surface reconstruction. *Neuroimage* **9**, 179–194 (1999).

25. Fischl, B. & Dale, A. M. Measuring the thickness of the human cerebral cortex from magnetic resonance images. *Proc Natl Acad Sci U S A* **97**, 11050–11055 (2000).

26. Fischl, B. *et al.* Whole brain segmentation: automated labeling of neuroanatomical structures in the human brain. *Neuron* **33**, 341–355 (2002).

27. Desikan, R. S. *et al.* An automated labeling system for subdividing the human cerebral cortex on MRI scans into gyral based regions of interest. *NeuroImage* **31**, 968–980 (2006).

28. Fortin, J.-P. *et al.* Harmonization of cortical thickness measurements across scanners and sites. *NeuroImage* **167**, 104–120 (2018).
